# Chiropractors in Finland – a demographic survey

**DOI:** 10.1186/1746-1340-16-9

**Published:** 2008-08-27

**Authors:** Stefan Malmqvist, Charlotte Leboeuf-Yde

**Affiliations:** 1 Ruoholahden Kiropraktikkokeskus (Private practice), Itämerenkatu 12, 00180 Helsinki, Finland; 2 Nordic Institute for Chiropractic and Clinical Biomechanics, part of Clinical Locomotion Science, University of Southern Denmark, Denmark

## Abstract

**Background:**

The Finnish chiropractic profession is young and not fully accepted by Finnish healthcare authorities. The demographic profile and style of practice has not been described to date. However, as the profession seems to be under rapid development, it would be of interest to stakeholders, both chiropractic and political, to obtain a baseline description of this profession with a view to the development of future goals and strategies for the profession. The purpose of this study was to describe the chiropractic profession in Finland in relation to its demographic background, the demographics of their clinics, practice patterns, interactions with other health care practitioners and some of the professions' plans for the future.

**Methods:**

A structured questionnaire survey was conducted in 2005, in which all 50 members of the Finnish Chiropractic Union were invited to participate.

**Results:**

In all, 44 questionnaires were returned (response rate 88%). Eighty percent of the respondents were men, and 77% were aged 30 to 44 years old, most of whom graduated after 1990 with either a university-based bachelors' or masters' degree in chiropractic. Solo practice was their main practice pattern. The vast majority described their scope of practice to be based on a musculoskeletal approach, using the Diversified Technique, performing Soft Tissue Therapy and about two-thirds also used an Activator Instrument (mechanical adjusting instrument). The mean number of patient visits reported to have been seen weekly was 59 of which nine were new patients. Most practitioners found this number of patients satisfactory. At the initial consultation, 80% of respondents spent 30–45 minutes with their patients, 75% spent 20–30 minutes with "new old" patients and on subsequent visits 80% of respondents spent 15–30 minutes. Interactions with other health care professions were reasonably good and most of chiropractors intended to remain within the profession.

**Conclusion:**

The Finnish chiropractic profession is relatively young. Consequently, many of the practitioners have a university-degree, which reflects recent developments in undergraduate chiropractic education. Their practice profile and the manner in which they practice appear to be fairly traditional.

## Background

Finland is a country situated in the north of Europe, consisting of approximately 5 million inhabitants. The climate is characterized by cold winters and relatively warm summers. Most inhabitants, 95%, speak Finnish, a language that is difficult to learn for foreigners since its vocabulary lacks common roots with most other European languages, and has a structure that differs significantly from the classical languages. English was not taught as a foreign language to all primary school pupils until the early 1970s. As a result the Finnish people was not strongly influenced by the Anglo-American culture.

The previous main sources of income were forestry, agriculture, fishery and heavy industry. In the period between its liberation from Russia in 1917 until World War II, the economy was weak. Finland was deeply scarred by its participation in World War II, and the economy improved only slowly thereafter, partly due to the large post-war "fine" that had to be paid to the Soviet Union. Over the past decades, an advanced electronic industry has developed and much of the rural population has become urbanized. The standard of living is now high, as is the educational standard, and much emphasis is put on various public health measures [1-3].

Traditionally, folk medicine has played a large role, and still does, particularly among people in the rural areas. It is therefore not surprising that Finland was late to "discover" chiropractic. Not until in the early 1950s did the first North American-educated chiropractor set up practice, followed by a second chiropractor 20 years later. Subsequently, the profession grew very slowly with competition from folk healers and manual therapists, who were typically trained through weekend courses.

Attempts have been made to ensure a world-wide minimum standard for all chiropractic institutions, through the use of an eductional control organisation, the Council on Chiropractic Eduction (CCE). There are branches in different parts of the world, and the European branch (ECCE) inspects and certifies all European chiropractic institutions, whether private or state funded. Chiropractic educational institutions that have achieved full certifiction by the ECCE are the Anglo-European College of Chiropractic, UK, the University of Glamorgan, UK, l'Institut Franco Européen de Chiropratique, France, and the University of Southern Denmark, Denmark. There is no ECCE-approved chiropractic education in Finland, so a chiropractic degree must be obtained in foreign countries.

During the late 1980s and early 1990s legislation for chiropractors was introduced in the other Nordic countries. In Finland, a new law introduced in 1994 licenced chiropractors with an academic degree from foreign chiropracic educational institutions, and grants were made available for students to study chiropractic abroad. The legal situation for the chiropractic profession has improved, but the working conditions are still unsatisfactory. Although the professional title recently became protected by law, chiropractors are unable to refer patients to other health care providers, cannot perform their own radiological examination, do not have direct access to imaging services, and may not prescribe sick leave. Unlike in the other Nordic countries, chiropractic patients in Finland are not entitled to government-subsidized reimbursement.

Despite this situation, the size of the Finnish Chiropractic Union membership has, according to personal communication with its secretariat, increased five-fold during the last 15 years. To date there are, according to communication with the Finnish National Authority for Medicolegal Affairs, 70 chiropractors in Finland with either a DC degree or an academic chiropractic degree, of which 52 (Jan 2008) are members of the Finnish Chiropractic Union.

It was the purpose of this study to describe the chiropractic profession in Finland, in terms of demographic and educational background. The demographics of their clinics included location, practice pattern, interactions with other health care practitioners, and some of the chiropractors' plans for the future.

## Methods

A survey was conducted using a structured questionnaire [Additional file 1: Demographic questionnaire for chiropractors in FCU]. All chiropractors who at the time of the study were members of the Finnish Chiropractic Union (N = 50) were invited to participate. The selection of participants was limited to members of the Finnish Chiropractic Union to ensure participation of graduates from CCE/ECCE accredited educational institutions.

Appropriately qualified graduates who were non-members of the Finnish professional association were not approached because they, from experience, are unwilling to participate in any communal activities. The questionnaire was first tested in a pilot study on 10% of the members for face validity, and the main survey was then administered in June 2005.

Approval was sought from the Helsinki University Ethics Committee, but because the survey was considered a quality assurance project approval was not needed. However, all questionnaires were coded to avoid recognition of respondents and the code key was destroyed when data collection was completed. Return of questionnaire implied consent from the participant. In order to respect the anonymity of the participants, in such a small group of practitioners, no comparison was made between responders and non-responders.

Data were entered into the SPSS 11.0 spreadsheet by a person experienced in data entry. Eleven questionnaires were randomly selected and each item was manually checked versus the entered data. No errors in the data entry were identified, which was considered satisfactory.

Therefore it was not considered necessary to undertake a double entry.

Analysis was done using SPSS 11.0 and Minitab. Some of the variables were grouped into fewer categories, based on the frequency of responses. The results were reported as descriptive data in tables and summarized in the text.

## Results

### Description of study sample

Forty-four of the 50 distributed questionnaires were returned, a response rate of 88%. Eighty percent of the respondents were men and 77% were aged 30 to 44 years [Additional file 2]. Eighty percent practised in a city suburb or city center. Forty-eight percent had one practice only, followed by 40% with two practices, and 12% with more than two practices. However, about one third expected to enter a partnership with a colleague within the next two years. Forty-five percent worked together (in the same clinic) with another health care provider and another 25% expected to do so within two years. Two of the respondents expected not to be working as a chiropractor at that time.

Fourteen percent employed a (non-chiropractic) assistant and a further 23% expected to do so within the next two years. About half had access to a receptionist. The majority (77%) graduated between 1990 and 2004 and 53% reported to have practised actively for a maximum of 10 years [Additional file 3]. Only 32% had a diploma of Doctor of Chiropractic, the remaining had either a university-based bachelor or master degree. Nine percent would consider undertaking an additional university degree. In addition, almost half of the members subscribe to a professional journal, usually the Journal of Manipulative and Physiological Therapeutics. In relation to interactions with other health care professions, these seem to be reasonably good [Table 1]. For example, the mean number of conversations/phone calls with other health care personnel in the past week was 3.3.

**Table 1:** A description of professional interactions between 44 Finnish chiropractors and other health care practitioners.

Variables	Subgroups	Frequency	Percentage
Received at least one referral last week from...	Medical practitioner	28	64
	Physiotherapist	12	27
	Masseur	23	52

Sent at least one report in relation to referral last week	Yes	18	41
	No	26	59

Had at least one conversation/phone call with other health care personnel last week	Yes	23	52
	No	21	48

Quality of co-operation with other health care providers	Mainly good	21	48
	Both good and bad	12	27
	Mainly lack of co-operation	11	25

### Scope of practice

The survey instrument also included questions on scope of practice, type of technique used, and adjunctive therapies used [Additional file 4]. The vast majority described their scope of practice to be based on a musculoskeletal approach. Almost all used the Diversified Technique, the vast majority performed Soft Tissue Therapy, and about two-third also made use of an Activator Instrument.

Various adjunctive therapies were used but none of these was used by all or even by the majority. Ice was most commonly reported, by 46%. Seventy-seven percent had a viewing box for radiology readings, 40% had the possibility to, indirectly, refer patients for X-ray examination via medical practitioners, of which 9% could refer for MRI or CT scans, whereas the use of ultrasound was very rare.

### Patients

The mean and median patient numbers, respectively, during the third week of 2005 was reported to be 59 and 47. However, there was a wide range, from 5 to 228. The mean and median number of new patients in that week was 9 and 2, respectively, indicating that the spread of data were skewed. The number of new patients in the past week preceding the study was also stated to be 9.

Eighty percent of the participants spent 30–45 minutes with their patients at the first visit, 75% spent 20–30 minutes on "new old" patients, whereas in subsequent visits 80% of respondents spent 15–30 minutes. At one extreme, one respondent reported spending one minute only on subsequent visits. The number of patient seen wasconsidered to be "about right" by 55%, and 9% reported they were seeing more patients than they would like to. However, about one third (36%) would have been happy to see some more patients.

Eighty percent had the (mandatory) malpractice insurance, 73% had a private pension scheme and almost as many (68%) had a private health care insurance.

## Discussion

The Finnish Chiropractic profession is relatively young and small, compared to other national chiropractic associations in Europe, and the demographics and practice procedures of the profession have never previously been documented [4]. Perhaps for this reason, Finnish chiropractors were eager responders to this survey with 88% returning the questionnaire.

According to the present survey, the members of the Finnish Chiropractic Union consisted of mainly young men (80%), who, in the majority of cases, graduated from university based or university affiliated chiropractic institutions. Information acquired from the administrative offices of corresponding associations in Sweden, Norway and Denmark reveals a different gender distribution, with 70%, 71% and 51%, respectively of men in the three countries. The proportion of male practitioners was lower (63%) also in a recent study of German chiropractors (response rate 72%) [5].

Despite the young age of the Finnish chiropractors, their current practice pattern was similar to that of the early years of chiropractic in Finland. Typically a Finnish chiropractor is working in solo practice, sharing his time between one or two practices. Sixty percent reported working in a solo practice, whereas, according to a previous study (response rate 70%), the estimated proportion was 41% among European chiropractors in general [6]. In the more recent German study, 45% of the respondents worked in a solo practice setting [5].

The Finnish Chiropractic Union subscribe to the Chiropractic Report for its members, a cost that is included in the membership fee. Additionally does almost 50% of the members subscribe to one more professional scientic journal. The Finnish chiropactor thus seem to be academically updated. However, regarding future development is only a small number interested in further education at a university level. This is understandable at the present time, considering the isolated position of the chiropractic profession, and the absence of chiropractic academic institutions in Finland. The time, money and effort spent on further education, would lead to no additional career possibilities.

Respondents were satisfied with the number of patients and they seemed to enjoy reasonable contacts with other health care practitioners. This may indicate that their professional activities are felt to be fulfilling. Nevertheless, two of the respondents were planning to leave the profession, although they were not close to retirement age.

Most reported to have a musculoskeletal approach, using mainly Diversified Manipulation Technique, Soft Tissue Techniques and Activator Instrument. These are methods previously reported frequently to be used in Europe [5-7].

The use of adjunctive therapies showed a less distinct pattern, perhaps because chiropractors determined that different patients require different approaches. It was also interesting that about one-third of the respondents had some sort of rehabilition equipment in their clinic, indicating that they also have the facility to assist patients with general or specific training following the acute treatment stage.

Regarding professional activities, only some of our data are comparable with information from previous European surveys, such as time spent with patients. The time spent on the first visit appeared to be similar in Finland and the Netherlands (36 and 41 minutes, respectively) [8]. Subsequent visits took 22 minutes in Finland and 15 minutes in the Netherlands.

Most of the Finnish chiropractors had made sure that they were covered with insurances both for pension scheme and private healthcare but, a small number appeared not to have the obligatory malpractice insurance.

The limitations of this study are that, despite the high response rate, not all chiropractors with a CCE/ECCE-approved education are members of the Finnish Chiropractors' Union and that not all members of the professional association participated in the survey. It is possible that non-participants in the study have a profile that differs from that of the responders. Other limitations are, of course, that the questionnaire was not exhaustive. For example, the description of practice procedures might have been designed differently by other groups of researchers, and the participants were not encouraged to extend their answers beyond the the questions stated in the questionnaire. Therefore, it is possible that some nuances of clinical practice failed to be recorded. However, the results of the pilot testing of the survey instrument did not indicate that the questionnaire failed to provide meaningful answering options.

## Conclusion

The educational background of the chiropractic participants in this study reflects the recent development in chiropractic education, with university affiliations and masters degrees. Although the Finnish chiropractic profession is relatively young, these chiropractors appeared to have a traditional practice profile: solo practice, a musculoskeletal approach, allowing good time for examination and treatment.

## Competing interests

The authors declare that they have no competing interests.

## Authors' contributions

SM was responsible for planning and executing the demographic survey, participated in the data collection and drafted the manuscript. CL–Y supervised the process. Both SM and CL–Y participated in the design of the study and performed the analysis. Both authors read, finalized and approved the final manuscript.

## Supplementary Material

Additional file 1 Demographic questionnaire for chiropractors in FCU. A translation of the original Finnish questionnaire. Click here for file

Additional file 2 Table 2. Description of 44 Finnish chiropractors and their practice patterns, I. Click here for file

Additional file 3 Table 3. Description of 44 Finnish chiropractors and their practice patterns, II. Click here for file

Additional file 4 Table 4. A description of scope of practice, techniques and adjunctive therapies used according to a survey of 44 Finnish chiropractors. Click here for file
