# Supplementary material for: Chiropractors in Finland – a demographic survey
Source: Chiropr Osteopat. 2008 Aug 27;16:9. doi: 10.1186/1746-1340-16-9 (PMC2535588; doi:10.1186/1746-1340-16-9)
Supplement: Additional file 1 — Demographic questionnaire for chiropractors in FCU. A translation of the original Finnish questionnaire. [file 1746-1340-16-9-S1.doc]

Demographic questionnaire for chiropractors in FCU

Tick appropriate box when needed.

**GENERAL INFORMATION**

**1. Sex:** Male Female

**2. Your age:**

<25  25-29

30-34  35-39

40-44  45-49

50-54  55-59

60-64  65-69

**3. Where is your main practice located?**

Countryside

Village

Small town

City, suburb

City, centre

**4. In which period did you graduate?**

1970 – 74

1975 – 79

1980 – 84

1985 – 89

1990 – 94

1995 – 99

2000 – 04

**5. At which level is your chiropractic academic degree?**

DC  Bachelor  Master  Other specify:_______________________

**6. I have been working as a chiropractor, at least half time, for ____ years in total**

**7. How many practices, and type of practice(s), do you have at the moment?**

| No. | Solo practice | Plural practice | Multidisciplinary |
| --- | --- | --- | --- |
| 1 |  |  |  |
| 2 |  |  |  |
| 3 |  |  |  |
| 4 |  |  |  |

**8. Do you have a receptionist?**

No  Yes, it is my own receptionist  Yes, I share receptionist with others

**INTERACTION WITH OTHER HEALTHCARE PROFESSIONS**

**9. Last week, how many referrals did you have and from whom?**

Number: ___ from GP

___ from MD, speciality _________________

___ from MD, speciality _________________

___ from MD, speciality _________________

___ from MD, speciality _________________

___ from physiotherapist

___ from OMT physiotherapist

___ from masseur

___ from other healthcare provider(s), specify type of provider:__________________

**10. Did you send any referral reports last week to health care personnel**?

No Yes if yes, please specify number_____

**11. How many conversations/phone calls did you have last week with any healthcare**

**personnel (Other than chiropractors) regarding your patients?** Number_____

**12. Do you feel that you have good cooperation with other healthcare providers?**

Mainly good cooperation Both good and bad  Mainly lack of cooperation

**TREATMENT & EXAMINATION**

**13. What is your scope of practice? If more than one – mark in numbering order.**

Subluxation based practice

Rehab practice

Wellness

Consultant

Acupuncture

Mainly musculoskeletal problems

Occupational health (ergonomics)

Cognitive Behavioural Therapy

Counselling on diet/weight

Other: ________________________________________

**14**. **What type of technique(s) do you use?**

Manipulation

- Diversified
- Gonstead
- Toggle

- Other: ________________________________________

Activator

Massage

Other soft tissue work

TMJ

Applied Kinesiology

SOT

Other: ________________________________________

**15.a. What type of tools/equipment do you use in your treatment?**

Laser

Electrotherapy-equipment

Ultrasound

Hydroculation

Ice/cool spray

TENS

Orthotics

Rehab tools (rocker board, gym balls etc.)

Gym equipment (on the premises)

Gym equipment (external)

Other: ________________________________________

**15.b. What type of table do you use in your treatment?**

Stationary table

Electric table

Toggle table

Knee-Chest table

Drop-piece table

Cox table

Levander table

Other: ________________________________________

**15.c. Do you have a reading box for radiological images?**

Yes  No

**16. How much time do you usually spend on a patient?**

1^st^ consultation: _____minutes

Subsequent standard consultations: _____minutes

Return of old patient with new problem: _____minutes

**17. Do you have the possibility to radiologically examine your patient?**

Yes  No

If yes, specify:

Plain x-ray

CT

MRI

Ultrasound

Other: ________________________________________

**PATIENT NUMBERS**

**18. How many patient visits did you have in the third week of 2005?**

Total number of visits ______, of which ______were new patients.

(I was not working the third week, but report from ______week instead)

**19a. In the past month, how do you feel about your patient numbers?**
 I have been rather too busy

It has been about right

I would have been happy to see some more patients

**19b**.

In your last full working week, how many new patients did you then have? ______

**INSURANCE**

**20. What kind of insurance(s) do you have?**

Private pension scheme/insurance Yes  No

Private healthcare insurance Yes  No

Private accident/malpractice insurance Yes  No

**FUTURE**

**21. Within the next two years, how likely do you think it is that you will …**

**a. employ a chiropractic assistant?**
Not very likely  Quite likely  Don't know  Already employed

**b.** **work together with (as a partner in the same clinic) another chiropractor (other than now)?**

Not very likely  Quite likely  Don't know

**c. work together with (in the same clinic) one or several other healthcare providers?**

Not very likely  Quite likely  Don't know  Already do

**d. have begun further education for a higher academic degree in chiropractic?**

Not very likely  Quite likely  Don't know  Already begun

Specify:……………

**e. not be working as a chiropractor?**Not very likely  Quite likely  Don't know

**OTHER**

**22. Do you subscribe to a professional journal?**

Yes  No   If yes, which one? ………………………………………………….
