# Supplementary material for: Chiropractors in Finland – a demographic survey
Source: Chiropr Osteopat. 2008 Aug 27;16:9. doi: 10.1186/1746-1340-16-9 (PMC2535588; doi:10.1186/1746-1340-16-9)
Supplement: Additional file 2 — Table 2. Description of 44 Finnish chiropractors and their practice patterns, I. [file 1746-1340-16-9-S2.doc]

## Table 2 - Description of 44 Finnish chiropractors and their practice patterns, I.

| **Variable Subgroups** | | **Frequency** | **Percentage** |
| --- | --- | --- | --- |
| **Gender** | Male  female | 35  9 | 80  20 |
| **Age** | 25-29  30-34  35-39  40-44  45-64 | 4  10  13  11  6 | 9  23  30  25  13 |
| **Location** | Countryside/Village | 2 | 4 |
|  | Small town | 7 | 16 |
|  | City suburb/centre | 35 | 79 |
| **No. of single practices** | 1 | 21 | 48 |
|  | 2 | 18 | 41 |
|  | 3 | 5 | 11 |
| **Sharing practice within next 2 yrs** | Not very likely | 19 | 43 |
|  | Quite likely | 13 | 29 |
|  | Don’t know | 10 | 23 |
|  | No reply | 2 | 4 |
| **Work in multidisciplinary practice within next 2 years** | Not very likely | 9 | 20 |
|  | Quite likely | 10 | 23 |
|  | Don’t know | 4 | 9 |
|  | Already do | 20 | 45 |
|  | No reply | 1 | 2 |
| **Change profession within next 2 yrs** | Not very likely | 41 | 93 |
|  | Quite likely | 2 | 4 |
|  | Don’t know | 1 | 2 |
| **Employ assistant within next 2 years** | Not very likely | 23 | 52 |
|  | Quite likely | 10 | 23 |
|  | Don’t know | 4 | 9 |
|  | Already employed | 6 | 14 |
|  | No reply | 1 | 2 |
