# Supplementary material for: Chiropractors in Finland – a demographic survey
Source: Chiropr Osteopat. 2008 Aug 27;16:9. doi: 10.1186/1746-1340-16-9 (PMC2535588; doi:10.1186/1746-1340-16-9)
Supplement: Additional file 3 — Table 3. Description of 44 Finnish chiropractors and their practice patterns, II. [file 1746-1340-16-9-S3.doc]

**Table 3 - Description of 44 Finnish chiropractors and their practice patterns, II**.

| **Receptionist** | No | 18 | 41 |
| --- | --- | --- | --- |
|  | Yes, it is my own | 12 | 27 |
|  | Share with others | 12 | 27 |
|  | No reply | 2 | 4 |
| **Year of graduation** | <1975 | 1 | 2 |
|  | 75-79 | 1 | 2 |
|  | 80-84 | 3 | 7 |
|  | 85-89 | 5 | 11 |
|  | 90-94 | 10 | 23 |
|  | 95-99 | 8 | 18 |
|  | 00-04 | 16 | 36 |
| **Years in practice** | 0-3 | 10 | 23 |
|  | 4-9 | 13 | 30 |
|  | >10 | 19 | 43 |
|  | No reply | 2 | 4 |
| **Qualifications** | DC | 14 | 32 |
|  | BSc | 18 | 41 |
|  | MSc | 12 | 27 |
| **Continuing higher degree** | Not very likely | 30 | 68 |
|  | Quite likely | 3 | 7 |
|  | Don’t know | 9 | 20 |
|  | Already begun | 1 | 2 |
|  | No reply | 1 | 2 |
| **Subscribe to a professional journal** | Yes | 18 | 41 |
|  | No | 22 | 50 |
|  | No Reply | 4 | 9 |
