# Supplementary material for: Chiropractors in Finland – a demographic survey
Source: Chiropr Osteopat. 2008 Aug 27;16:9. doi: 10.1186/1746-1340-16-9 (PMC2535588; doi:10.1186/1746-1340-16-9)
Supplement: Additional file 4 — Table 4. A description of scope of practice, techniques and adjunctive therapies used according to a survey of 44 Finnish chiropractors. [file 1746-1340-16-9-S4.doc]

**Table 4 - A description of scope of practice, techniques**

**and adjunctive therapies used according to a survey**

**of 44 Finnish chiropractors**.

| **Scope of practice** | **Frequency** | **Percent** |
| --- | --- | --- |
| Subluxation based | 22 | 50 |
| Musculoskeletal approach | 38 | 86 |
| Rehabilitation | 14 | 32 |
| Wellness | 21 | 48 |
| Chiropractic consulting | 8 | 18 |
| Occupational health care | 9 | 21 |
| Acupuncture | 3 | 7 |
| Diet | 7 | 16 |
| **Technique** |  |  |
| Diversified | 43 | 98 |
| Gonstead | 16 | 36 |
| Toggle | 21 | 48 |
| Activator | 30 | 68 |
| Soft tissue therapy | 37 | 84 |
| Massage | 12 | 27 |
| Applied kinesiology | 19 | 43 |
| SOT | 16 | 36 |
| Other | 10 | 23 |
| **Adjunctive therapies** |  |  |
| Ice | 20 | 46 |
| Orthotics | 12 | 27 |
| Rehabilitation equipment | 15 | 34 |
| Gym (on premises) | 2 | 4 |
| Gym (external) | 9 | 20 |
| Electrotherapy | 2 | 4 |
| Ultrasound | 2 | 4 |

**More than one reply possible**
